# The role of extracellular vesicle-derived miRNAs in adipose tissue function and metabolic health

**DOI:** 10.1097/IN9.0000000000000027

**Published:** 2023-07-26

**Authors:** Bam D. Paneru, David A. Hill

**Affiliations:** 1 Division of Allergy and Immunology, Department of Pediatrics, Children’s Hospital of Philadelphia, Philadelphia, PA, USA; 2 Department of Pediatrics, Institute for Immunology, and Institute for Diabetes, Obesity and Metabolism, Perelman School of Medicine, University of Pennsylvania, Philadelphia, PA, USA

**Keywords:** obesity, miRNA, endocrine, paracrine, extracellular vesicle, exosome

## Abstract

Extracellular vesicles (EVs) are nanometer size lipid particles that are released from virtually every cell type. Recent studies have shown that miRNAs carried by EVs play important roles in intercellular and interorgan communication. In the context of obesity and insulin resistance, EV-derived miRNAs functionally bridge major metabolic organs, including the adipose tissue, skeletal muscle, liver, and pancreas, to regulate insulin secretion and signaling. As a result, many of these EV-derived miRNAs have been proposed as potential disease biomarkers and/or therapeutic agents. However, the field’s knowledge of EV miRNA-mediated regulation of mammalian metabolism is still in its infancy. Here, we review the evidence indicating that EV-derived miRNAs provide cell-to-cell and organ-to-organ communication to support metabolic health, highlight the potential medical relevance of these discoveries, and discuss the most important knowledge gaps and future directions for this field.

## 1. Introduction

White adipose tissue (AT) is a highly adaptable tissue that can change its physiology and function to maintain metabolic homeostasis in the face of insufficient or excessive caloric intake. However, extreme and protracted caloric intake can also lead to AT dysfunction that is characterized in part by insulin resistance, inadequate energy storage/inappropriate lipolysis, and inflammation ^[1]^. Decades of research have shown that this dysfunction is central to the development of other obesity-associated sequela such as type 2 diabetes mellitus (T2DM), steatohepatitis, and cardiovascular disease ^[2,3]^. As a result, there is considerable focus on identifying therapeutic strategies and medications that maximize and extend the normal functions of AT as a means of maintaining metabolic health and avoiding the later-stage health consequences of obesity.

The fate of AT during obesity is determined in part by extensive communication that occurs between adipocytes and adipose tissue macrophages (ATMs) ^[4]^. Recently, extracellular vesicles (EVs) have been identified as a key mechanism of adipocyte-ATM communication ^[5–10]^. EVs are a heterogenous group of membrane-bound vesicles released from cells which can be broadly classified into two main categories: exosomes (50–150 nm) and microvesicles (50–1000 nm) ^[11]^. Microvesicles are formed and released from the cell by outward budding of the plasma membrane, whereas exosomes are formed within multivesicular bodies (MVB) by inward budding of the MVB membrane, with subsequent release from the cell upon fusion of the MVB membrane with the plasma membrane ^[11]^. After release from the parent cell, EVs enter into interstitial space and ultimately into circulation where they can be taken up by distantly located tissues and cells, thereby exerting systemic effects. EVs carry various lipids, proteins, RNAs, and DNA from the cell of origin ^[12]^ which mediate the functions of EVs in intercellular communication ^[13]^.

miRNAs are the most studied EV cargo in various disease contexts, including oncologic, cardiovascular, neurological, and infectious diseases ^[14–18]^. miRNAs are small noncoding RNAs that bind to the 3’UTR of target mRNAs, leading to suppression of translation and/or degradation of the target mRNA ^[19]^. The result is negative regulation of the expression of the gene product. miRNAs are very potent gene regulators, as a single miRNA can target hundreds of genes ^[20]^. Recent studies suggest that miRNAs are responsible for many of the identified EV effects in the context of obesity and metabolism ^[5]^. Notably, MiRNA profiles of secreted EVs depend on cell type and the physiological status of the cell. In addition, the packaging of miRNAs into EVs does not appear to be random, as specific sets of miRNAs can be sorted into EVs via the recognition of miRNA sequence motifs by sorting proteins ^[21–24]^. Thus, the secretion of miRNAs via EV is a regulated process that likely evolved to mediate important intercellular and interorgan coordination and maintain metabolic homeostasis.

Emerging studies suggest that EV-mediated transfer of miRNAs in an endocrine or paracrine manner may be central to multiple aspects of mammalian metabolism that extend beyond adipocyte-macrophage interaction and AT functions ^[12]^. The goal of this review is to summarize the most recent knowledge about EV miRNA-mediated regulation of mammalian metabolism, discuss the potential for these discoveries to impact medical care, and emphasize knowledge gaps to delineate the future research directions needed to advance the field. First, we will review and discuss how EV miRNA-mediated adipocyte-ATM crosstalk in AT determines the fate of AT function and systemic metabolism. Next, we will summarize and discuss endocrine-like long-range communications mediated by EV miRNAs between various metabolic organs. Then, we will review and discuss the therapeutic and biomarker potentials of EVs and EV miRNAs. Finally, we will discuss the limitations of current studies and will discuss future research directions related to the successful use of EV and EV miRNAs in disease diagnosis and therapy.

## 2. Regulation of adipocyte functions by macrophage-derived EV miRNAs

ATMs are critical regulators of AT function during obesity ^[25]^. Traditionally, ATMs are known to regulate AT functions by numerous mechanisms, such as by producing biologically active cytokines and clearing dead adipocytes and their lipid remains via phagocytosis ^[26]^. Recently, ATMs have been shown to also influence adipocyte and AT functions by delivering miRNA into adipocytes (**Table 1**) ^[5–9]^. The earliest in-depth study to show the significance of EV-mediated delivery of miRNA from ATMs to adipocytes in vivo came from Ying et al ^[6]^. In this study, the authors transfected bone marrow-derived macrophages (BMDM) with Cy3-labeled miR-223 and then co-cultured BMDM and 3T3-L1 adipocytes in a transwell plate. After 12 hours, a robust Cy3 signal was observed in 3T3-L1 adipocytes confirming the delivery of Cy3-miR-223 from the BMDMs to the 3T3-L1 adipocytes. In another experiment, they purified EVs from ATMs of obese mice, labeled the EVs with red dye PKH26, and then added the labeled EVs into the 3T3-L1 culture. After incubation, EVs, together with their miRNA cargo, were readily taken up by 3T3-L1 adipocytes, as evidenced by the acquisition of red PKH26 color and EV miR-223 by 3T3-L1 cells. Finally, the authors co-cultured ATMs and 3T3-L1 adipocytes in a transwell plate with or without the EV-secretion inhibitor GW4869 to establish that miR-223 is delivered from ATMs into 3T3-L1 adipocytes via EVs. Together, these experiments established for the first time that ATMs can secrete EVs containing miRNA cargo that can be taken up by recipient adipocytes.

**Table 1.** Metabolically important miRNAs secreted into EVs by different cell types and receptor cells, target gene, and metabolic function of these miRNAs.

Cell secreting EVs	Recipient tissue/cell	miRNA in EV	miRNA target	miRNA function	Reference
ATMs (obese mice)	Adipocyte, myocyte, and hepatocyte	miR-155	*Ppar-γ*	Induces insulin resistance	^[6]^
ATMs (obese mice)	Adipocyte, myocyte, and hepatocyte	miR-29a	*Ppar-δ*	Induces insulin resistance	^[7]^
M2 polarized BMDMs (in vitro)	Adipocyte, myocyte, and hepatocyte	miR-690	*Nadk*	Promotes insulin signaling	^[5]^
Kupffer cell (normal lean mice)	Hepatocyte, hepatic stellate cells	miR-690	*Nadk*	Counteracts NASH (nonalcoholic fatty acid steatosis) liver phenotypes	^[27]^
Macrophage (Raw264.7) (in vitro cultured in high glucose media)	Adipocytes	miR-210	*Ndufa4*	Induces insulin resistance	^[9]^
Skeletal muscle (during high-intensity exercise)	Liver cells	miR-133a, miR-133b	*Foxo1*	Maintain metabolic homeostasis	^[28]^
T lymphocyte (nondiabetic obese/prediabetic mice)	Pancreatic β cell	miR-142-3p, miR-155, miR-142-5p	Not determined	Promote β cell apoptosis and may contribute to type 1 diabetes development	^[29]^
Hepatocyte (early onset obesity)	AT, skeletal muscle, and liver cells	miR-3075	*Fa2h*	Augments insulin signaling	^[30]^
Islet resident M1 macrophage (Obese mice)	Pancreatic β cell	miR-212-5p	*Sirt2*	Inhibits insulin secretion	^[31]^
Pancreatic β cells (lean mice)	Hepatocyte	miR-26a		Augments insulin sensitivity	^[32]^
Skeletal muscle	Adipose tissue	miR-146a-5p	GD5F	Inhibits adipogenesis	^[33]^

ATM, adipose tissue macrophages; BMDM, bone marrow-derived macrophages; EV, extracellular vesicle.

ATM populations include diverse groups of macrophages with distinct functions ^[26]^. For example, ATMs in the lean state have a predominantly anti-inflammatory phenotype (characterized as “M2” activation in many studies), which are thought to primarily promote normal AT homeostasis and function. In contrast, ATMs in obese AT have a predominantly proinflammatory phenotype (often characterized as “M1” activation), which are thought to contribute to AT dysfunction and metabolic disease. Originally, these contrasting functions of ATM subsets were thought to be mediated mainly by the ATM cytokine profile as well as by differences in the ATM’s ability to phagocytose dead adipocytes and scavenge and metabolize lipids ^[26]^. However, recent works show that ATM functions are also supported by distinct miRNA secretomes. For example, Ying et al ^[6]^ purified EVs from the ATMs of obese mice and injected them intravenously into lean mice for 2 weeks. EV administration induced AT and systemic insulin resistance, an effect that is consistent with the presiding functions of obesity-associated ATMs. The authors showed that this phenotype was mediated predominantly by miR-155, which was overexpressed in obese ATM EVs and targeted the transcription factor *Ppar-γ* in adipocytes. In a separate study, Liu et al ^[7]^ also showed that the administration of obese ATM-derived EVs to lean mice causes insulin resistance, an effect that was attributed to miR-29a.

Complementing these studies, EVs secreted by ATMs derived from lean mice attenuate insulin resistance and AT inflammation, and improve systemic glucose homeostasis, when administered to insulin-resistant obese mice ^[6]^. A similar effect was obtained by administering EVs derived from M2-polarized BMDMs ^[5]^. Notably, when EVs were purified from M2 polarized Dicer knockout BMDMs (which are miRNA-deficient), the effects of EV treatment on insulin resistance and glucose homeostasis were blunted, indicating miRNAs contribute to the observed effects. The authors went on to show that EVs derived from M2 polarized macrophages contain a high level of miR-690, a miRNA that targets *Nadk*, providing a potential mechanism by which M2 EVs may regulate insulin signaling in adipocytes. Finally, Tian et al ^[9]^ cultured Raw264.7 macrophages in vitro under normal or high glucose conditions to resemble normal physiological conditions and diabetic hyperglycemia scenarios, respectively. When this group treated 3T3-L1 adipocytes with EVs, they found that EVs isolated from high glucose but not from normal glucose BMDMs inhibited insulin signaling in 3T3-L1 cells. High glucose treatment of Raw264.7 was found to cause elevated expression of miR-210 in cells and their EV fractions, which inhibits the *Ndufa4* gene to regulate insulin signaling in recipient adipocytes ^[9]^. The above observations show that EVs and their miRNA cargo secreted by ATMs play an important role to determine adipocyte functions in AT in the context of obesity.

## 3. Regulation of macrophage functions by adipocyte-derived EV miRNAs

Extreme and persistent caloric intake puts considerable stress on AT and promotes a dramatic phenotypic switch in ATMs ^[34]^. Recently, a novel sub-class of ATMs, characterized by surface expression of CD9 (and labeled lipid-associated macrophages [LAMs]), has been identified as the predominant ATM present in obese AT of mice and humans ^[35,36]^. LAMs have a distinct transcriptional, metabolic, and functional profile and are beneficial to metabolic health early in obesity but adopt a proinflammatory phenotype later in the disease progression. Though cytokines and other factors that drive recruitment and phenotypic switches in ATMs during obesity have been identified ^[26]^, how lipid-laden hypertrophic and hydrophobic adipocytes influence ATM biology and function is less well understood. Recently, it has been appreciated that adipocytes also secrete large quantities of miRNA-loaded EVs during obesity ^[37]^, raising the possibility that adipocytes communicate with ATMs via EV miRNAs. For example, a recent study by Pan et al ^[10]^ indicates that adipocyte expression of miR-34a progressively increases with obesity in mice. The authors find that adipocyte-derived miR-34a is delivered to ATMs via EVs, where it inhibits *Klf4* and “M2” polarization, resulting in a more proinflammatory ATM phenotype.

While there are no additional studies at this time that demonstrate the regulation of ATM functions by the direct action of adipocyte-delivered EV miRNAs, several miRNAs are known to regulate aspects of ATM biology via cell-intrinsic targeting of ATM genes. Some such examples include regulation of Notch signaling and inflammatory function by miR-30 ^[38]^, inhibition of M1 proinflammatory function by miR-99a by targeting tumor necrosis factor alpha (TNFα) ^[39]^, potentiation of activation by miR-125b by inhibiting interferon regulatory factor 4 (IRF4) ^[40]^, inhibition of proinflammatory cytokine production by miR-124 by targeting signal transducer and activator of transcription 3 (STAT3) and TNF-α–converting enzyme (TACE) ^[41]^, promotion of M2 and inhibition of M1 polarization by miR-125a-5p by inhibiting Krüppel-like factor 4 (KLF4) ^[42]^, inhibition of nuclear factor kappa-light-chain-enhancer of activated B cells (NF-κB) pathway by miR-146a by inhibiting interleukin 1 receptor associated kinase 1 (IRAK1) and TNF receptor associated factor 6 (TRAF6) ^[43]^, promotion of proinflammatory phenotype by miR-127 by targeting B-cell lymphoma 6 (BCL6) ^[44]^, inhibition of M2 and promotion of M1 response by miR-155 by targeting CCAAT enhancer binding protein beta (C/EBPβ) and suppressor of cytokine signaling 1 (SOCS1) ^[45,46]^, let-7c mediated inhibition of M1 polarization and activation of M2 polarization by targeting p21-activated kinase 1 (PAK1) and CCAAT enhancer binding protein delta (C/EBP-δ) ^[47,48]^, and inhibition of M2 polarization by miR-1224 by inhibiting Musashi RNA binding protein 2 (MSI2) ^[42]^. Of these miRNAs, Zhang et al ^[49]^ show that mature adipocytes from mice release miR-1224 into exosomes, which when co-cultured with BMDM in vitro, deliver miR-1224 into BMDM to inhibit M2 polarization. Similarly, Thomou et al ^[37]^ found that adipocyte-specific deletion of Dicer leads to at least a 7-fold decrease in the level of miR-99a, miR-30, miR-146a, let-7c, and miR-155 in the blood, suggesting that these miRs are secreted into EVs by adipocytes. Thus, it is likely that adipocytes deliver these miRNAs into neighboring ATMs to regulate ATM functions—an axis that should be investigated further.

## 4. Other paracrine and endocrine-like functions of EV miRNAs in metabolism

Endocrine-like functions of EV-secreted miRNAs are made possible through the release of EVs into the blood circulation and other body fluids, from where they subsequently target cells in distant tissues and organs. The first in vivo demonstration of long-range gene regulation via EV miRNAs came from a study by Thomou et al ^[37]^. In this study, the authors cloned human miR-302f (which does not have a murine homolog) into an adenoviral vector and injected the recombinant virus directly into the brown adipose tissue (BAT) of mice to achieve BAT-specific miR-302f expression. They then cloned the miR-302f target 3’UTR downstream of a luciferase reporter into another adenoviral vector and injected it intravenously into the same mice, resulting in the expression of the construct in the liver. The authors observed a 95% reduction in luciferase expression in the liver of mice injected with miR-302f compared with the control lacZ vector, indicating that miR-302f from BAT had been transferred to the liver. To confirm that miR-302f transfer from BAT to the liver was delivered via EVs, the authors isolated serum EVs from mice expressing miR-302f in BAT and transferred them into mice expressing the target 3’UTR luciferase construct in the liver. As expected, EV administration suppressed liver luciferase expression by ~95% in the recipient mice. Thus, these experiments provided the first evidence of endocrine-like, long-range communication between two different metabolic organs mediated by an EV-resident miRNA.

**Figure 1.**
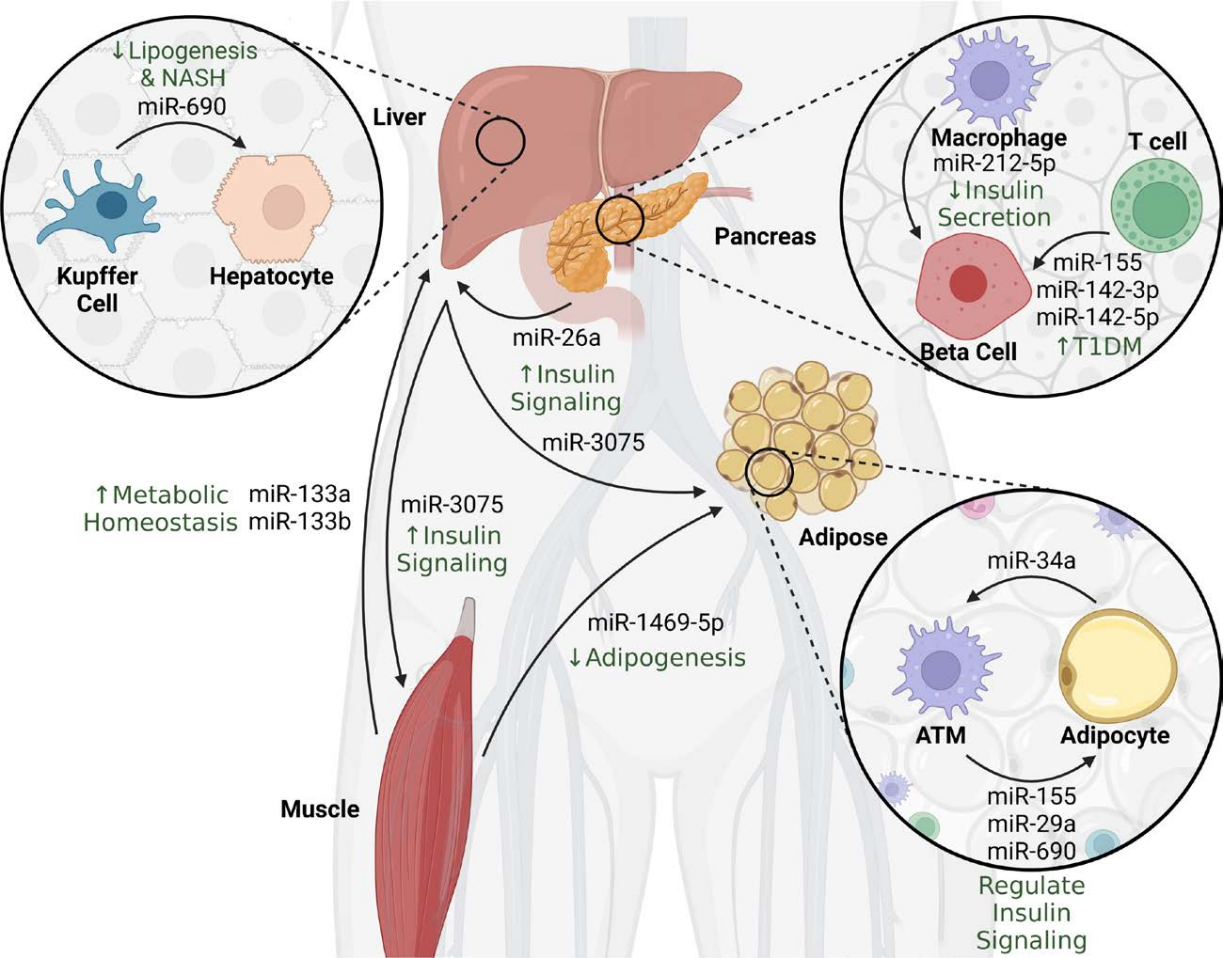
**Summary of metabolically relevant miRNAs and their originating and targeting cells and/or organs.** The overall effect of miRs is in green. NASH, nonalcoholic steatohepatitis; T1DM, type 1 diabetes mellitus.

Recently, several other examples of interorgan communication by EV miRNAs have been discovered in the context of obesity and metabolism. For example, skeletal muscle releases EVs containing several metabolically important miRNAs into blood circulation ^[50]^. The miRNA profile of skeletal muscle-secreted EVs varies with the physiological and disease stage of the animal, suggesting that EV miRNA cargo may serve as a mediator of the underlying physiology. As an example, high-intensity interval training (HIIT) in mice increases the level of miR-133 in EVs secreted by skeletal muscle ^[28]^. When these EVs are administered to sedentary mice, they improve metabolic homeostasis, mimicking the beneficial effects exercise has on metabolic health. This effect is thought to occur primarily via the inhibition of hepatic *Foxo1* by EV-delivered miR-133 ^[28]^. On the other hand, hepatocytes release miR-3075-enriched EVs during early onset obesity (4 weeks of high-fat diet), which when injected into insulin-resistant recipient mice, attenuate insulin resistance. This function of hepatic EVs on insulin signaling is believed to occur as a result of miR-3075s action on recipient extrahepatic tissues such as adipose tissue and skeletal muscle ^[30]^. In another study, Qin et al ^[33]^ show that miR-146a-5p is highly enriched in skeletal muscle-derived EVs. When these EVs are injected into recipient mice, exosomes, together with miR-146a, are readily taken up by various adipose tissue depots, where miR-146a-5p inhibits adipogenesis ^[33]^. Similarly, Xu et al ^[32]^ report that miR-26a not only regulates insulin secretion by acting on pancreatic β-cells but also regulates insulin signaling in peripheral tissues such as the liver via EV-mediated transfer. These examples suggest that EV miRNAs bridge metabolic organs to provide metabolic coordination in the context of normal physiology as well as obesity and insulin resistance (Figure 1).

In addition to insulin signaling in peripheral tissues, insulin secretion by the pancreas is also regulated by EV miRNAs. Qian et al ^[31]^ show that M1 macrophages, which accumulate in the pancreas during obesity and contribute to β-cell dysfunction, act via the release of miR-212-5p-enriched EVs. When the authors treated pancreatic islets with M1 macrophage-derived EVs in vitro or directly injected EVs into recipient mice via pancreatic ductal infusion, EVs were readily taken up by pancreatic islets. EV treatment resulted in the inhibition of insulin secretion by pancreatic islets both in vitro and in vivo, which may be attributed to the direct inhibition of the *Sirt2* gene by miR-212-5p contained in the EVs ^[31]^. A similar study by Guay et al ^[29]^ demonstrates that T cell infiltration of pancreatic islets that contributes to the selective elimination of insulin-secreting β-cells in the context of type I diabetes mellitus (T1DM) is mediated by EVs secreted by T cells. The authors show that miR-142-3p, miR-142-5p, and miR-155 are upregulated in EVs secreted by T cells of nondiabetic obese mice, a model of T1DM. When these EVs are co-cultured with pancreatic islets in vitro, EVs together with their miRNA cargo are taken up by the islets where they trigger β-cell apoptosis potentially by regulating cytokine and chemokine signaling pathways. The above observations demonstrate the regulation of both insulin secretion and insulin action by EV-mediated miRNA delivery in a paracrine and endocrine manner.

## 5. EVs and EV miRNAs as potential biomarkers and therapeutic agents

There is considerable data in preclinical animal models to suggest promising therapeutic potential for EVs and EV miRNAs in the treatment of metabolic diseases. Some such examples include the treatment of obesity-associated sequela ^[5]^ and nonalcoholic fatty acid steatosis (NASH) ^[27]^ in mice by miR-690 mimic, treatment of obesity and its sequela in mice by EVs derived from skeletal muscle of HIIT mice ^[28]^, and treatment of type 1 diabetes by EVs derived from mesenchymal stem cells in rats ^[51]^. Circulating EV miRNAs have also been proposed as disease biomarkers for various metabolic diseases ^[52–57]^. This is relevant as miRNAs can be detected with greater specificity and simplicity by techniques such as reverse transcription-polymerase chain reaction (RT-qPCR) compared with antigen-antibody reactions used for protein biomarkers and colorimetric/fluorometric reactions used for lipid biomarkers. Efforts to clinically validate miRNA biomarkers show promising specificity and sensitivity for the diagnosis and monitoring of metabolic diseases ^[58]^. As an example, serum levels of miR-133a and miR-1 are associated with the diagnosis of T2DM with sensitivity of 78.9% and 78.9%, respectively, and specificity of 74.2% and 71.0%, respectively ^[58,59]^. Similarly, three urinary EV miRNAs (miR-133b, miR-342, and miR-30a) have a specificity of 72.7%, 80.9%, and 90.9%, respectively, and a sensitivity of 86.4%, 81.8%, and 76.4%, respectively, for detecting type 2 diabetic nephropathy ^[58,60]^. Furthermore, serum levels of EV miRNA miR-29a and miR-29b have a sensitivity of 76.47% and 85.29%, respectively, and a specificity of 78.18% and 81.82%, respectively, in detecting gestational diabetes. When the expression level of miR-29a and miR-29b are combined, the sensitivity and specificity climb to 86.76% and 90.91%, respectively ^[61]^. Though knowledge about the function of these miRNAs in the context of metabolic diseases is still primitive, diverse potential functions have been reported for miR-133a (adipose tissue browning ^[62]^, diabetic cardiomyopathy ^[63]^, myoblast proliferation ^[64]^, and osteoblast differentiation ^[65]^), miR-133b (renal fibrosis ^[66]^ and neurite growth ^[67,68]^), miR-1 (myogenesis ^[64]^, angiogenesis ^[69]^, and synaptic function ^[70]^), miR-342-5p (heart function ^[71]^, neural stem cell proliferation ^[72]^, and osteoblasts proliferation, differentiation, and migration ^[73]^), miR-30a (adipose tissue remodeling ^[74]^ and autophagy in the context of ischemic heart disease ^[75]^), miR-29a (insulin signaling ^[7]^, potential β-cell insulin secretion ^[76]^, hepatocyte function and liver fibrosis ^[77]^, and angiogenesis and osteogenesis ^[78]^), and miR-29b (aging-associated insulin resistance ^[79]^, potential β-cell insulin secretion ^[76]^, osteoblast differentiation ^[80]^, and muscle atrophy ^[81]^). Thus, natural EVs and EV-derived miRNAs have promising clinical applications as both therapeutics and biomarkers.

## 6. Future research directions

Together, the above observations show that miRNA cargo of ATM and other cell-secreted EVs plays important roles in mediating obesity and metabolic homeostasis. However, studies involving ATMs and AT biology to date have generally considered all ATMs in either lean or obese AT to be homogeneous. They have also neglected to consider miRNA or EV heterogeneity within the secretome of a particular ATM subset. Finally, we have a poor understanding of how the whole ATM miRNA secretome may work antagonistically or synergistically to cause a particular effect during a given disease state. As such, future studies should focus on the characterization of EV miRNA profiles from distinct ATM subpopulations, the study of EV and miRNA heterogeneity within a single ATM secretome, and ultimately the integration of multiple miRNA effects at early and late timepoints in the obesity progression to identify key determinants of the overall ATM functional program.

One of the challenges of using miRNAs as therapeutic agents lies in the delivery of the miRNA molecules in vivo. Several miRNA vehicles, such as synthetic liposomes and different types of polymeric vehicles, have been used to deliver miRNAs ^[82]^. However, these delivery agents lack tissue targeting specificity and are not effective at delivering miRNAs into certain difficult-to-transfect cells and tissues. Since EVs serve as a natural vehicle for miRNA cargo, natural or engineered EVs could serve as a better alternative. EVs utilize several surface membrane proteins for their fusion and internalization by the target cell ^[83]^, which could be exploited to provide for targeted miRNA delivery to specific organs or tissues. Indeed, there is some evidence that EV uptake by recipient cells can be cell type specific ^[84,85]^. In the cancer field, Nie et al ^[86]^ have utilized this organotrophic feature of breast cancer cell-derived EVs to deliver miR-126 to nonsmall cell lung cancer cells. More recently, researchers have been trying to engineer EVs by genetic or chemical modification of surface proteins in such a way that EVs are recognized and taken up by only specific cell subsets ^[87]^. As an example, engineered exosomes in which the surface protein Lamp2b is fused with the neuron-specific rabies virus glycoprotein peptide efficiently and specifically deliver miRNA and siRNA to the brain ^[88,89]^. Similarly, a genetically engineered exosome in which EV surface protein CD63 is fused with ApoA-1 delivers miRNA selectively and efficiently to liver cells ^[90]^. These examples suggest that EVs could be engineered, at least by modification of the surface protein, to facilitate selective uptake by metabolic organs such as the liver, AT, muscle, and pancreas for therapeutic intervention. However, future research should focus on identifying molecules that are specifically recognized by the cell surface receptors of metabolic organs, generating engineered EVs by fusion of these molecules with relevant EV surface molecules, and testing the tissue targeting ability of such engineered EVs in the context of metabolic diseases.

For successful use of circulating EV miRNAs as disease biomarkers, first, we must determine the complete EV miRNA profile dysregulated during the disease. Results from current diagnostic approaches that rely on the detection of a single miRNA are difficult to interpret as one miRNA can have an association with multiple physiological processes ^[91]^. As described above and observed by Deng et al ^[61]^, the combination of multiple miRNAs would improve the specificity and sensitivity of the diagnosis. Second, EVs are purified from body fluids by using several different techniques, such as differential ultracentrifugation, poly-ethylene glycol-based precipitation, size-exclusion chromatography, antibody-based immunoaffinity capture, and microfluidic devices, resulting into different levels of purity and functional integrity of EVs ^[92]^. More importantly, these techniques yield a highly heterogenous EV population released from multiple tissues ^[93]^, and the inclusion of nonrelevant tissue EVs may compromise the diagnostic accuracy. Even antibody-based methods currently used to isolate EVs rely on common EV surface markers such as CD9, CD63, and CD81 ^[92]^, which are present on EVs released from virtually every tissue. One approach to developing tissue-specific EV purification from the circulation could be the use of an antibody-based method to target EV membrane proteins that are specific to their parental cells. Recently, Sun et al ^[94]^ have reported the successful creation of an EV capture chip device based on a cocktail of antibodies that target multiple hepatocellular carcinomas (HCC)-associated membrane proteins. Using this method, they report promising selective enrichment of HCC-associated EVs from an artificially pooled EV cocktails as well as from patient serum ^[94]^. Thus, future studies should focus on the identification and characterization of such tissue-specific marker proteins in the context of metabolic diseases to facilitate tissue-specific EV enrichment for metabolic diseases. Such approaches will further strengthen the sensitivity and specificity of diagnostics and biomarkers, as only EVs from relevant tissue(s) will be interrogated.

## 7. Conclusion

EV miRNA-mediated cell-to-cell communication in paracrine and endocrine manner plays an important role in maintaining metabolic homeostasis. However, our understanding of EV miRNA biology in the context of mammalian metabolism is rapidly evolving. Future research progress in fields such as the characterization of complete EV miRNA transcriptome of relevant metabolic tissues, fractionation of tissue-specific EVs from the circulation, and engineering of EVs to use as tissue-targeted miRNA drug delivery vehicles will determine the true potential of EV and EV miRNAs in clinical applications.

## Conflicts of interest

The authors declare no conflicts of interest.

## Funding

Immunometabolic and obesity-related research in the Hill Lab is supported by the NIH (K08 DK116668, R03 DK129418, and R01 HL162715 to DAH).
